# Raising public awareness of glaucoma in Ethiopia

**Published:** 2012

**Authors:** Abeba T Giorgis

**Affiliations:** Consultant senior ophthalmologist and glaucoma specialist, Department of Ophthalmology, School of Medicine, Addis Ababa University, Addis Ababa, Ethiopia. Email: abebatgiorgis@yahoo.com

**Figure:**
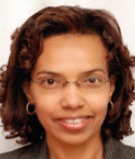
Abeba T Giorgis

In Ethiopia, glaucoma is the fifth most common cause of blindness and the disease caused irreversible blindness in an estimated 62,000 people in 2006.^1^

Due to the nature of the disease, an inadequate and inaccessible eye care service, and a very poor level of public awareness, glaucoma patients tend to come for help after they have become either unilaterally or bilaterally blind.

Even among some health professionals in Ethiopia, awareness and understanding of glaucoma is low. There are many instances of parents being told that their child does not have an eye problem when in fact they are suffering from congenital glaucoma, and I have seen many people with acute angle-closure glaucoma who have been treated for conjunctivitis!

In 2007, a group of volunteers made up of physicians and glaucoma patients set up the Glaucoma Group, with the aim of increasing public awareness of glaucoma. The group is supported by the Ophthalmological Society of Ethiopia (OSE) in collaboration with the Department of Ophthalmology, Addis Ababa University, and the Ministry of Health.

The mass media has been the key to the success of the group's activities as it is a means of reaching millions of people. The Glaucoma Group has worked with the media on documentaries, discussion programmes, and short ‘advertising’ type messages on both television and radio.

The first glaucoma message was broadcast on television in 2008 and was paid for by Light for the World. There were also programmes and educational messages on national and regional radio stations. We approached the media through the OSE and through Menelik Hospital, where the Department of Ophthalmology is based. The people working in the media were interested in broadcasting health-related programmes and were very helpful.

The Glaucoma Group produced posters, banners, brochures, leaflets, a book about glaucoma translated into Amharic, caps, and T-shirts, which were given to glaucoma patients and their families.

**Figure:**
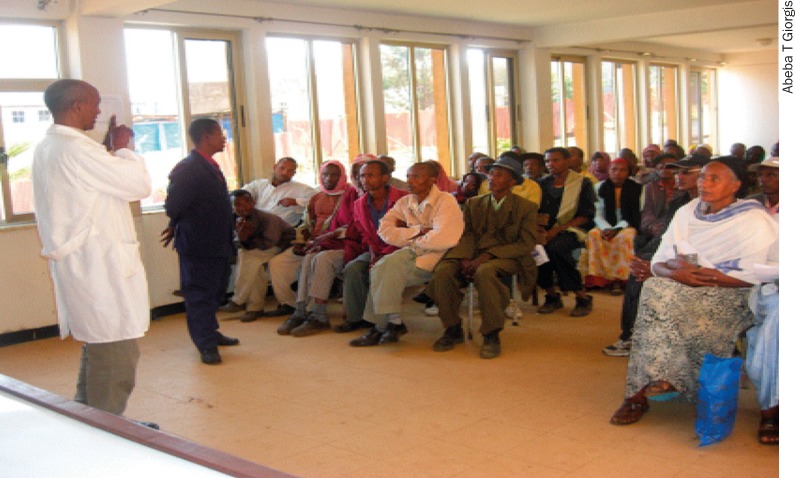
A glaucoma education session for patients and their families at Jimma University Eye Centre. ETHIOPIA

Here are a few examples of the key messages the group communicated through the media at different times.

- ‘Did you know there is an eye disease called ‘glaucoma’ (related to high eye pressure) which is different from trachoma?’ (this was the first message, in 2008).
- ‘Did you know that glaucoma could steal your sight?’
- ‘You have a high chance (likelihood) of developing glaucoma if you have parents, sisters, or brothers living with glaucoma.’
- ‘If you have glaucoma, encourage your family to be checked for glaucoma to prevent your loved ones from losing vision due to glaucoma blindness.’
- ‘Using steroid eye drops for a long time increases your risk of glaucoma and cataract.’

**Figure:**
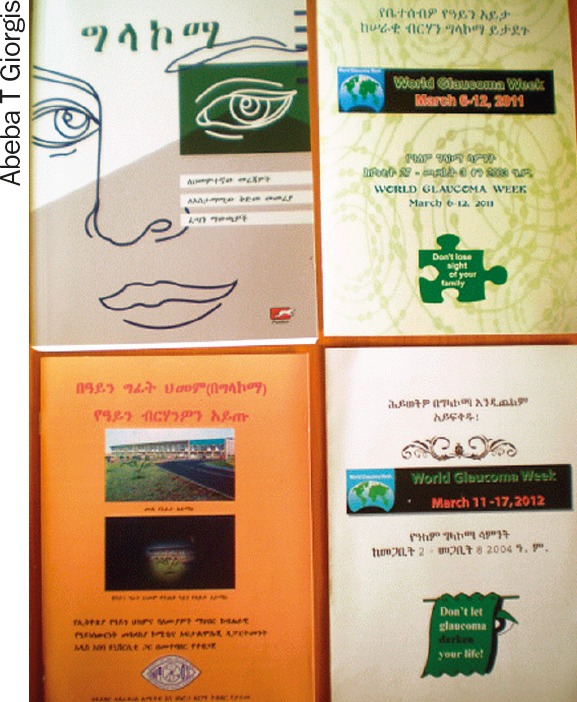
Translated glaucoma book and brochures in Amharic

Every year, during World Glaucoma Week, trained nurses also provided glaucoma education to patients, relatives, and carers at ophthalmic centres in Addis Ababa, Jimma, and Gondar. The OSE approached drug distributors to fund these activities. The translation of the glaucoma book to Amharic, and the printing costs, were paid for by the author, Josef Flammer of Basel Eye Hospital in Switzerland.

**‘The level of glaucoma awareness has increased from 4% to 28%’**

Since these public awareness activities began, most opthalmologists practising in Ethiopia have noticed an increase in the number of people who come to be examined for glaucoma. Many patients now ask ophthalmologists and ophthalmic nurses whether they are being checked for glaucoma, even if they have come for some other reason (such as getting a prescription for reading spectacles). It has also become quite common to see siblings and children of glaucoma patients coming forward to be examined.

The level of glaucoma awareness among ophthalmic patients at the same tertiary eye care centres has increased from 4% in 2006 to 28% in 2011 (research not yet published). The proportion of patients coming for follow-up appointments, and the numbers who accept medical and surgical treatment, have also increased. Eye care providers have become more aware of glaucoma, some as a result of seeing or hearing something in the mass media, and others because of the questions patients ask them.

Raising public awareness of glaucoma is a key means of addressing this devastating eye condition. In our experience, using the mass media is the easiest and most effective way to do so.
